# Methods for Analytical Validation of Novel Digital Clinical Measures: Implementation Feasibility Evaluation Using Real-World Datasets

**DOI:** 10.2196/70314

**Published:** 2025-11-17

**Authors:** Simon Turner, Lysbeth Floden, Leif Simmatis, Piper Fromy, Joss Langford, Eric J Daza, Andrew Potter, Kathleen Troeger, the STAGES cohort investigator group

**Affiliations:** 1 Digital Medicine Society, Boston, MA, United States; 2 Quantitative Science, Evinova, 35 Gatehouse Drive, Waltham, MA, 02451, United States, 1 (520) 360-3962; 3 Department of Speech-Language Pathology, Temerty Faculty of Medicine, University of Toronto, Toronto, ON, Canada; 4 Seeing Theta, Saumur, France; 5 Activinsights Ltd., Kimbolton, Cambridgeshire, United Kingdom; 6 Department of Public Health and Sport Sciences, University of Exeter, Exeter, United Kingdom; 7 Stats-of-1, Menlo Park, CA, United States; 8 Division of Biometrics I, Office of Biostatistics, Center for Drug Evaluation and Research, US Food and Drug Administration, Silver Spring, MD, 20993, United States; 9 Stanford University, Stanford, CT, United States

**Keywords:** digital health technologies, analytical validation, digital medicine, confirmatory factor analysis, novel digital clinical measures

## Abstract

**Background:**

Sensor-based digital health technologies (sDHTs) are increasingly used to support scientific and clinical decision-making. The digital measures (DMs) they generate offer significant potential to accelerate the drug development timeline, decrease clinical trial costs, and improve access to care. However, choosing an appropriate statistical methodology when conducting analytical validation (AV) of a DM is complicated, particularly for novel DMs, for which appropriate, established reference measures (RMs) may not exist. More understanding of, and a standardized approach to, AV in these scenarios is needed.

**Objective:**

In a prior simulation study, 3 statistical methods were tested for their ability to estimate a simulated relationship between a sDHT-derived DM and several clinical outcome assessment (COA) RMs. The aim of this work was to assess the feasibility of implementation of these methods in real data and to examine the impact of AV study design factors on the relationships estimated.

**Methods:**

Four real-world datasets, captured using sDHTs, were used to prepare hypothetical AV studies representing a range of scenarios with respect to 3 key study design properties: temporal coherence, construct coherence, and data completeness. The datasets analyzed were as follows: Urban Poor (comparing nighttime awakenings to measures of psychological well-being), STAGES (comparing daily step count to psychological and fatigue measures), mPower (comparing daily smartphone screen taps to measures of function in Parkinson’s disease), and Brighten (comparing smartphone communication activity to measures of psychological well-being). For each hypothetical AV study, 3 statistical methods were leveraged: the Pearson correlation coefficient (PCC) between DM and RM, simple linear regression (SLR) between DM and RM, multiple linear regression (MLR) between DMs and combinations of RMs, and 2-factor, correlated-factor confirmatory factor analysis (CFA) models. Performance measures were the PCC magnitudes (for PCC), R^2^ and adjusted R^2^ statistics (for SLR and MLR, respectively), and factor correlations (for CFA).

**Results:**

Most of the CFA models exhibited an acceptable fit according to the majority of the fit statistics employed, and each model was able to estimate a factor correlation. For each model, these correlations were greater than or equal to the corresponding PCC in magnitude. Correlations were the strongest in the hypothetical studies with strong temporal and construct coherence.

**Conclusions:**

The performance of the selected statistical methods shown in this work supports their feasibility when implemented in real-world data. Our findings, in particular, support the use of CFA to assess the relationship between a novel DM and a COA RM. The observed impact of AV study design factors on the relationships estimated allowed the authors to determine practical recommendations for study design in AV of novel DMs. By using a standardized methodology for evaluating novel DMs, sDHT developers, biostatisticians, and clinical researchers can navigate the complex validation landscape more easily, with more certainty, and with more tools at their disposal.

## Introduction

Sensor-based digital health technologies (sDHTs) are increasingly used to support scientific and clinical decision-making. The digital measures (DMs) they generate offer significant benefits, including the potential to accelerate the drug development timeline, decrease clinical trial costs, and improve access to care [1]. This potential has motivated considerable efforts to expand research into the application of novel digital measures to capture clinically relevant data and establish endpoints that the community has previously been unable to assess using traditional methods of data collection and statistical analysis [2,3].

A novel digital measure can be defined as either a measure that has not previously been assessable or an existing measure that is being applied in a new population, environment, or context of use.

The evaluation of the digital measures derived from sDHTs as fit for purpose is the first step in bringing the value of these technologies to the people who can benefit the most. The well-established V3+ framework [4] and its recent extension to include usability [5] provide a robust, modular framework for developers and regulators to follow when evaluating measures generated from sDHTs. The V3+ framework states that to support scientific and clinical decision-making, investigators must undertake verification of the sensor(s), usability validation of the sDHT, analytical validation (AV) of any algorithm(s) applied, and clinical validation of a measure of a clinical or functional state in a defined context of use.

AV represents a critical bridge between initial technology development (ie, verification) and clinical utility (ie, clinical validation). An AV study comprises reporting on the comparison between the output of a novel sDHT’s algorithm and 1 or more reference measures (RMs).

While work exists that has developed standardized methodology for clinical validation [6], the same methodology development and standardization is now required for AV. Of note, the difficulty in defining the performance requirements and in selecting the appropriate statistical methodology to assess against these requirements is of premier importance.

This difficulty is magnified when working with novel sDHTs for which appropriate, established RMs may not exist or may have limited applicability. For an example of this limitation, in speech, articulatory function assessed via digital audio recordings is a relatively straightforward measure to analytically validate because there are existing high-quality RMs that can form the basis of comparisons [7]. However, for digital cognitive assessments, such comparisons may not be so straightforward as existing RMs may be restricted to instruments such as clinical outcome assessments (COAs) that capture multiple aspects of disease severity as a single semiquantitative score [8]. The issue here is that the output of the sDHT and the RM does not directly correspond in such situations. This means that traditional analyses such as receiver operating characteristic curves and intraclass correlations are often not possible.

In a prior simulation study [9], several statistical methods were tested for their ability to return a nonbiased estimate of the simulated relationship between an sDHT-derived DM and COA RMs. Simulation studies provide evidence for the feasibility of the methods in ideal situations; however, in data collected in practice, in either clinical or real-world settings, nuances can lead to issues such as model nonconvergence. Here, we examine the implementation of the methods previously examined in simulation, across several real-world datasets with varying data missingness, sample size, and theoretical relationship between the DM and RM. The aim of this work was to assess the feasibility of the methods’ implementation in real data and to examine the impact of AV study design factors on the relationships estimated. As with the prior simulation study [9], COAs were used as the RMs in order to evaluate AV study design factors, to reflect situations where they comprise the only available RMs and thus represent the measurement target of interest.

## Methods

### Selection of Datasets

Four open-access datasets were employed for this research; the Urban Poor dataset [10,11], the STAGES dataset [10], the mPower dataset [12], and the Brighten dataset [13]. These datasets were selected based on several preferred characteristics:

- At least 100 subject records (repeated measures were permitted)
- Data captured using a sDHT
- At least one sDHT variable (acting as the digital measure) that was:
  - Collected on seven or more consecutive days
  - A discrete variable, aggregated as an ordinal variable representing a record of events occurring
  - Either available as, or able to be summarized as, a daily summary format (eg, number of steps per day)

- COAs to act as RMs that:
  - Assessed a similar construct to the sDHT variable(s)
  - Assessed each item on a Likert scale
  - At least 1 COA with a daily recall period and at least 1 COA with a multiday recall period
    - A COA with a daily recall period asks a participant to consider a single day when they answer, such as a global impression of severity [14]. Conversely, a COA with a multiday recall period asks a participant to consider more than 1 day; for example, the PHQ-9 [15] asks a participant to think about how they have felt over the preceding 2 weeks. All claims must be validated and verified and backed up with sufficient evidence (subject to regulatory review).

These characteristics were chosen to allow us to construct hypothetical AV studies in keeping with the V3+ framework, while respecting the prerequisite requirements for each chosen statistical method to function robustly. The 4 datasets selected represented a variety of quality in terms of key properties of an AV study design: temporal coherence, construct coherence, and data completeness (Textbox 1). The datasets selected also represent the best matches available that met most of the COA characteristics. Table 1 summarizes the key properties of each of the 4 selected datasets.

Textbox 1. **Analytical Validation Study Design Qualities.**

Certain aspects of study design offer the best opportunity to observe a relationship between a digital measure and a reference measure, where such a relationship exists.

These include the following:

- **Temporal coherence:** the similarity between the periods of data collection for the measures.
- **Construct coherence:** the similarity between the theoretical underlying constructs being assessed by the measures.
- **Data completeness:** the level of data completeness in both the digital measure and reference measure data. Study design should have a strategy to maximize data completeness.

**Table 1.** Summary of investigated datasets^a^

Title	Usable sample size	Digital measure(s)	Reference measure(s)	Coherence characteristics
Urban Poor	452	Number of awakenings during an entire night	Rosenberg Self-Esteem Scale [16] Generalized Anxiety Disorder Questionnaire (GAD-7) [17] Patient Health Questionnaire (PHQ-9) [15] Daily single-item patient global impression of happiness [11]	Weak construct coherence (digital measure of sleep, reference measures of psychological well-being) Weak temporal coherence (multiday recall reference measures collected at baseline, before digital measure data collection; interventional study creates a potential change in the state of the underlying construct being assessed)
STAGES	964	Daily step count	Fatigue Severity Score (FSS) [18] Generalized Anxiety Disorder Questionnaire (GAD-7) [17] Patient Health Questionnaire (PHQ-9) [15] Nasal Obstruction Symptom Evaluation (NOSE) [19]	Weak construct coherence (digital measure of physical activity, reference measures of fatigue, psychological well-being, and breathing obstruction). Weak temporal coherence (reference measures were collected at inconsistent times during the study with respect to the digital measure data collection).
mPower	1641	No. of smartphone screen taps during a daily tapping activity	Selected questions from the Movement Disorder Society Unified Parkinson Disease Rating Scale (UPDRS) [20] Parkinson Disease Questionnaire (shortened version) (PDQ-8) [21]	Moderate-to-strong construct coherence (all measures targeted Parkinson disease, but both reference measures had broader scope than the digital measure). Strong temporal coherence with minimal missing data.
Brighten	89	Three variables from daily passive smartphone communications data: Unique numbers from incoming calls Unique numbers from outgoing calls Unique numbers from texts received	Patient Health Questionnaire (PHQ-9) [15] Two-item daily version of the PHQ-9 (PHQ-2) [22]	Moderate-to-weak construct coherence (Data are not adjusted for a subject’s normal behavioral habits) Moderate-to-strong temporal coherence (digital measure data from the full recall period of the PHQ-9 were analyzed, although there was substantial reference measure data missingness).

a A full description of the datasets analyzed can be found in Multimedia Appendix 1. Some of the datasets did not meet all the preferred characteristics. The Brighten data have a usable sample size less than 100; while there is a sufficient sample size (accounting for repeated measures) reported in the original study [13], the distribution of data missingness led to excluding many records in our analysis. Furthermore, the STAGES and mPower data lack applicable reference measures with daily recall periods.

### Statistical Methods

#### Data Preparation

For each dataset, we prepared each measure’s data for analysis via the following steps. Each step involved selecting, subsetting, or otherwise processing data values.

#### Multiday Recall RM Data Selection

For each study participant, each RM administration instance (ie, instance of an RM being administered) was included for analysis and considered repeated measures. Thus, if a participant answered an RM 3 times during the study period, all 3 responses were used in analysis.

For each instance, the raw scores for the individual items were aggregated per participant by summing and then linearly scaling them to fit a scale ranging from 0 to 100. For example, the PHQ-9 measure is a 9-item PRO with each item response scored on a 0-3 scale [15]. For each participant, raw scores were first summed, and the result was multiplied by 100/27 (analogous to the process of converting a raw score to a percentage). RM data values already on a 0-100 scale were assumed to be ready for analysis and were not modified.

#### Digital Measure Data Selection

For each study participant and for each multiday recall RM instance, we analyzed digital measure data that corresponded to the recall period of the RM. For example, the PHQ-9 has a recall period of 2 weeks. Thus, if a participant answered the PHQ-9 on January 14, then only digital measure data values from January 1 to January 14 inclusive were used in the analysis.

From this subset of digital measure data, we selected the 7 days of data closest to the RM administration instance. The 7-day criteria have been shown to be sufficient to achieve reliable data across a spectrum of populations and contexts of use [23-25]. Continuing the above example, if digital measure data were captured on all 14 days of the PHQ-9 recall period, then the 7 days of data selected for analysis would be January 8-January 14. If fewer than 7 days of digital measure data were observed during the RM recall period, then all such days were used in the analysis; all data values on the remaining days were treated as missing.

#### Daily RM Data Selection

For each study participant, we analyzed daily RM data that corresponded to the selected digital measure data. Continuing the above example, the 7 or fewer days of daily RM data selected for this participant would come from the period of January 8-January 14 inclusive. If daily RM data were not recorded on some days in this window, then these data values were treated as missing.

#### Further Processing of the Digital Measure Data and Daily RM Data

To properly deploy the full range of statistical methods for modeling and factor analysis, data values of the digital and daily RMs needed to be aggregated to match the administration cadence of the multiday recall RMs. This was accomplished by calculating the mean of all observed data values at each administration instance of a multiday recall RM, for each participant.

Continuing the above example, we would calculate a study participant’s mean digital measure “score” (ie, mean data value) over the period of January 8-January 14, inclusive. Likewise, we would calculate the mean daily RM score from the same January 8-January 14 window.

### Data Analysis

Table 2 presents a summary of the statistical approaches used in this work.

**Table 2.** Summary of statistical methods and evaluation criteria.

Analysis	Type	Description	Evaluation criteria
PCC^a^	Correlation	PCC between DM^b^ and individual RMs^c^.	The magnitude and sign of the PCC.
SLR^d^	Regression	SLR between DM and individual RMs.	Coefficient of determination (R^2^).
MLR^f^	Regression	MLR between DM and combinations of individual RMs.	Adjusted coefficient of determination (R^2^).
CFA^e^	Factor analysis	Two-factor confirmatory factor analysis of combinations of DM and RM data, modeled with correlations between latent factors.	CFI,^g^ TLI^h^, RMSEA,^i^ SRMR^j^

a PCC: Pearson correlation coefficient.

b DM: digital measure.

c RM: reference measure.

d SLR: simple linear regression.

e CFA: confirmatory factor analysis.

f MLR: multiple linear regression.

g CFI: comparative fit index.

h TLI: Tucker–Lewis index.

i RMSEA: root mean square error of approximation.

j SRMR: standardized root mean square residual.

Pearson correlation coefficients (PCCs), confirmatory factor analysis (CFA), and linear regression were used to analyze each dataset, following the same methodology in each case. A full description of the data analysis methods can be found in Multimedia Appendix 2; a summary of the methods appears below.

In each dataset, PCCs were calculated between each digital measure and each multiday recall RM.

Two-factor, correlated-factor CFA models were created for each combination of digital measure and multiday recall RM. CFA was selected, given its ability to model measurement error more explicitly than PCC as well as its insensitivity to scale differences (due to factors being computed from correlations, removing the influence of input variable scale), which we anticipated may be a useful property when dealing with measures containing multiple items/measures collected across sessions. It is additionally able to handle a range of measurement units/data types (continuous, ordinal, etc), which makes it well-suited to the problem of dealing with questionnaire data as well as sensor-derived data [26,27]. The correlation between the factors was calculated and used as the estimate of the relationship between the DM and RM. Four model fit statistics were computed for each model: Comparative Fit Index (CFI), Tucker–Lewis Index (TLI), root mean square error of approximation (RMSEA), and standardized root mean square residual (SRMR). The fit statistics were evaluated against the following thresholds to determine if each model was an acceptable fit to the data [28,29]: CFI and TLI acceptable fit: values≥0.9, and RMSEA and SRMR acceptable fit: values<0.08.

Simple linear regression (SLR) models were created to model the relationship between the digital measures and each multiday recall RM. Multiple linear regression (MLR) models were created to model the relationship between each digital measure and every combination of daily and multiday recall RMs available. R^2^ values were calculated for each model.

All analyses were performed using R statistical software v4.1.2 [30] along with several additional packages. The additional packages include the following: dplyr, readxl, stringr, and lubridate for data preparation; and lavaan and tibble for data analysis.

All packages were used in their September 2024 latest versions.

### Ethical Considerations

This study is a secondary use of data that are publicly available and have undergone institutional review board (IRB) review(s). Brief details of data access and ethical reviews undertaken by the teams that prepared each dataset are provided below.

The Urban Poor dataset is licensed under CC0 1.0 (public domain). Participants in this study provided informed consent, including information on the specific data collection methods used. Hypotheses of the study were not shared with the participants, but participants were told that the study was described as work to understand the “difficulties underprivileged people in India face, and how these problems affect their lives.” [10,11].

Data from the STAGES dataset are published openly on the National Sleep Research Resource for commercial and noncommercial use by the STAGES study team. Data use agreements were sought by the STAGES study team with individual research institutions to ensure compliance with specific IRBs’ policies. Detailed ethics and consent procedures are available as part of the open data release package [10].

Coded data from the mPower dataset are published openly on Synapse. E-consent was obtained from study participants before analysis and data sharing, including a distinction between “narrow” data sharing (ie, with only the mPower study team) or openly among the broader research community. Ethical oversight of the study was provided by Western IRB [12].

Data from the Brighten dataset are publicly available via Synapse. Informed consent was obtained before enrollment in the study. Ethical approval for the original study data collection was obtained via the University of California (San Francisco) Committee for Human Research [13].

Additionally, no identification of individual participants is possible from our use of the datasets in our hypothetical AV studies.

## Results

The results are presented in two parts: first, the functioning of the methods, and, second, the results arising from those methods, ie, the relationships between the measures that were estimated.

### Functioning of the Methods

In each dataset, results were successfully obtained for each of the methods investigated, and, in particular, each of the CFA models converged, which indicates that our chosen models can be fitted to the data.

### CFA Model Fit

Using the thresholds of acceptable fit detailed above, the model fit statistics suggested that the models in the Urban Poor, STAGES, and mPower datasets had an acceptable fit (Tables3^5^). In the Brighten dataset, the fit statistics were less clear, returning a mixed acceptability of the fit between each of the 4 calculated fit statistics (Table 6).

**Table 3.** Urban Poor CFA fit statistics.

Reference measure	CFA^a^ model fit measure^b^			
	CFI^c^	TLI^d^	RMSEA^e^	SRMR^f^
Rosenberg^g^	0.913	0.900	0.081	0.079
GAD-7^h^	1.000	1.000	0.000	0.034
PHQ-9^i^	0.994	0.993	0.024	0.042

a CFA: Confirmatory Factor Analysis.

b CFI and TLI acceptable fit: ≥ 0.90, RMSEA and SRMR acceptable fit: < 0.08.

c CFI: Comparative Fit Index.

d TLI: Tucker-Lewis Index.

e RMSEA: Root Mean Square Error of Approximation.

f SRMR: Standardized Root Mean Square Residual.

g Rosenberg: Rosenberg Self-Esteem Scale.

h GAD-7: Generalized Anxiety Disorder Questionnaire.

i PHQ-9: Patient Health Questionnaire-9.

**Table 4.** STAGES CFA fit statistics.

Reference measure	CFA^a^ model fit measure^b^			
	CFI^c^	TLI^d^	RMSEA^e^	SRMR^f^
FSS^g^	0.997	0.996	0.223	0.043
GAD-7^h^	0.997	0.996	0.255	0.037
PHQ-9^i^	0.996	0.996	0.238	0.061
NOSE^j^	0.997	0.996	0.314	0.063

a CFA: Confirmatory Factor Analysis.

b CFI and TLI acceptable fit: ≥ 0.90, RMSEA and SRMR acceptable fit: < 0.08.

c CFI: Comparative Fit Index.

d TLI: Tucker-Lewis Index.

e RMSEA: Root Mean Square Error of Approximation.

f SRMR: Standardized Root Mean Square Residual.

g FSS: Fatigue Severity Score.

h GAD-7: Generalized Anxiety Disorder Questionnaire.

i PHQ-9: Patient Health Questionnaire-9.

j NOSE: Nasal Obstruction Symptom Evaluation.

**Table 5.** mPower CFA fit statistics.

Reference measure	CFA^a^ model fit measure^b^			
	CFI^c^	TLI^d^	RMSEA^e^	SRMR^f^
UPDRS^g^	1.000	1.004	0.000	0.060
PDQ-8^h^	0.957	0.953	0.067	0.088

a CFA: Confirmatory Factor Analysis.

b CFI and TLI acceptable fit: ≥ 0.90, RMSEA and SRMR acceptable fit: < 0.08.

c CFI: Comparative Fit Index.

d TLI: Tucker-Lewis Index.

e RMSEA: Root Mean Square Error of Approximation.

f SRMR: Standardized Root Mean Square Residual.

g UPDRS: Movement Disorder Society Unified Parkinson’s Disease Rating Scale (selected questions).

h PDQ-8: Parkinson’s Disease Questionnaire (shortened version).

**Table 6.** Brighten CFA fit statistics.^a^

Digital measure	CFA^b^ model fit measure^c^			
	CFI^d^	TLI^e^	RMSEA^f^	SRMR^g^
Unique numbers calls incoming	0.906	0.890	0.151	0.106
Unique numbers call outgoing	0.965	0.959	0.504	0.131
Unique numbers texts received	0.968	0.963	0.311	0.121

a All statistics use the Patient Health Questionnaire-9 reference measure.

b CFA: Confirmatory Factor Analysis.

c CFI and TLI acceptable fit: ≥ 0.90, RMSEA and SRMR acceptable fit: < 0.08.

d CFI: Comparative Fit Index.

e TLI: Tucker-Lewis Index.

f RMSEA: Root Mean Square Error of Approximation.

g SRMR: Standardized Root Mean Square Residual.

The results were examined in more detail. When assessed using the CFI, each CFA model in each of the 4 datasets had an acceptable fit.

When assessed using TLI, all the CFA models had an acceptable fit, except for one of the 3 models built for the Brighten data.

When assessed using SRMR, there was agreement with CFI and TLI in the Urban Poor and STAGES datasets—the fit was acceptable in each model in these datasets. However, when assessing the Brighten model, SRMR deemed each of the models to have an unacceptable fit, in contrast to the assessment from CFI and TLI. When assessing the mPower model, the UPDRS model had an acceptable fit, but the PDQ-8 model did not.

When assessed using RMSEA, each model in the STAGES and Brighten datasets had an unacceptable fit. In the Urban Poor dataset, the CFA models using GAD-7 and PHQ-9 as the RM were deemed to be an acceptable fit according to RMSEA; however, the model fit when using the Rosenberg Self-Esteem scale as the RM was unacceptable. In the mPower dataset, all models had an acceptable fit according to RMSEA.

### Relationships Estimated

#### Correlations

The magnitude of the calculated correlations (Tables7^10^) varied depending on the dataset and the choice of digital and RMs. In the Urban Poor data, all the estimated relationships were negligible (maximum magnitude 0.052, minimum magnitude 0.001); in the STAGES data, the magnitude of the relationships varied between 0.087 and 0.180. Larger relationships were observed in the Brighten data (maximum magnitude 0.175 and 0.340 for Pearson correlation and CFA correlation, respectively) and mPower data (maximum magnitude −0.329 for both types of correlation).

**Table 7.** Urban poor correlation values.

Reference measure	Pearson correlation	CFA^a^ factor correlation
Rosenberg^b^	0.001	−0.028
GAD-7^c^	−0.032	−0.052
PHQ-9^d^	−0.021	−0.022

a CFA: Confirmatory Factor Analysis.

b Rosenberg: Rosenberg Self-Esteem Scale.

c GAD-7: Generalized Anxiety Disorder Questionnaire.

d PHQ-9: Patient Health Questionnaire-9.

**Table 8.** STAGES correlation values.

Reference measure	Pearson correlation	CFA^a^ factor correlation
FSS^b^	−0.178	−0.180
GAD-7^c^	−0.087	−0.099
PHQ-9^d^	−0.161	−0.175
NOSE^e^	−0.109	−0.120

a CFA: Confirmatory Factor Analysis.

b FSS: Fatigue Severity Score.

c GAD-7: Generalized Anxiety Disorder Questionnaire.

d PHQ-9: Patient Health Questionnaire-9.

e NOSE: Nasal Obstruction Symptom Evaluation.

**Table 9.** mPower correlation values.

Reference measure	Pearson correlation	CFA^a^ factor correlation
UPDRS^b^	−0.329	−0.329
PDQ-8^c^	−0.299	−0.319

a CFA: Confirmatory Factor Analysis.

b UPDRS: Movement Disorder Society Unified Parkinson’s Disease Rating Scale (selected questions).

c PDQ-8: Parkinson’s Disease Questionnaire (shortened version).

**Table 10.** Brighten correlation values^a^

Digital measure	Pearson correlation	CFA^b^ factor correlation
Unique numbers calls incoming	0.024	0.213
Unique numbers call outgoing	0.175	0.340
Unique numbers texts received	0.037	0.147

a All statistics use the PHQ-9 reference measure.

b CFA: Confirmatory Factor Analysis.

In all scenarios, the CFA factor correlation was larger in magnitude than the Pearson correlation; this difference in magnitude was subtle in the Urban Poor set (where all relationships were negligible), the STAGES data (between 10% and 15% difference), and the mPower data (where despite the larger magnitude in relationships, the difference between the two correlation types was of a similar magnitude to the Urban Poor data). However, the difference in correlation magnitude was much more noticeable in the Brighten set; CFA factor correlation was at least twice as large as Pearson Correlation in every scenario.

#### Regressions

In the Urban Poor, STAGES, and Brighten datasets, the calculated R^2^ values (either standard or adjusted; ) Tables11^13^ were negligible. There was a trend for the R^2^ values to be greater in magnitude in the Brighten dataset than in the STAGES dataset, which were in turn generally greater than those exhibited in the Urban Poor dataset.

In the mPower dataset (Table 14), the R^2^ values were much larger in magnitude than in the other datasets, although still small in general, with values between 0.123 and 0.139.

**Table 11.** Urban Poor R^2^ values^a^

Regression model type	Reference measure(s) included in the regression model	R^2^ (standard or adjusted as appropriate)
SLR^b^	Rosenberg^c^	<<0.001
	GAD-7^e^	0.001
	PHQ-9^f^	0.001
MLR^d^	All weekly surveys	−0.005
	All + daily (mean values)	−0.003
	All + daily (individual days)	−0.005

a The daily survey is a single-item global impression of happiness.

b SLR: simple linear regression.

c Rosenberg: Rosenberg Self-Esteem Scale.

d MLR: multiple linear regression.

e GAD-7: Generalized Anxiety Disorder Questionnaire.

f PHQ-9: Patient Health Questionnaire-9.

**Table 12.** STAGES R^2^ values^a^

Regression model type	Reference measure(s) included in the regression model	R^2^ (standard or adjusted as appropriate)
SLR^b^	FSS^d^	0.030
	GAD-7^e^	0.006
	PHQ-9^f^	0.024
	NOSE^g^	0.009
MLR^c^	All	0.033

a No daily surveys are included.

b SLR: simple linear regression.

c MLR: multiple linear regression.

d FSS: Fatigue Severity Score.

e GAD-7: Generalized Anxiety Disorder Questionnaire.

f PHQ-9: Patient Health Questionnaire-9.

g NOSE: Nasal Obstruction Symptom Evaluation.

**Table 13.** Brighten R^2^ values^a^

Digital variable	Regression model type			
	SLR^b^	MLR^c^		
		Daily 1	Daily 2	Both dailies
Unique numbers calls incoming	0.039	0.022	0.060	0.053
Unique numbers call outgoing	0.041	0.036	0.057	0.045
Unique numbers texts received	0.001	−0.024	−0.016	−0.029

a All statistics use the PHQ-9 multiday recall reference measure. The two daily reference measures are the two individual questions isolated from the PHQ-2 (Patient Health Questionnaire-2), which assesses depression severity and was adapted to become a daily measure in this study.

b SLR: simple linear regression.

c MLR: multiple linear regression.

**Table 14.** mPower R^2^ values^a^

Regression model type	Reference measure(s) included in the regression model	R^2^ (standard or adjusted as appropriate)
SLR^b^	UPDRS^c^	0.131
	PDQ-8^d^	0.123
MLR^e^	All	0.139

a No daily surveys are included.

b SLR: simple linear regression.

c UPDRS: Movement Disorder Society Unified Parkinson’s Disease Rating Scale (selected questions).

d PDQ-8: Parkinson’s Disease Questionnaire (shortened version).

e MLR: multiple linear regression.

In each dataset with a daily RM available (Urban Poor and Brighten), it was generally true that including daily RM data resulted in a stronger adjusted R^2^ than when not including it. In datasets without a daily RM (STAGES and mPower), using multiple RMs generally resulted in a stronger R^2^ than when using a single RM.

## Discussion

### Principal Findings

In this work, we assessed the feasibility of selected statistical methodology to estimate relationships between digital measures and COA RMs. We also investigated how properties of an AV study’s design may affect the strength of the estimated relationships by using several statistical methodologies. We accomplished this by using real-world data, captured using sensor-based digital health technologies, to conduct hypothetical AV studies across a range of scenarios.

Our analysis of the 4 real-world datasets demonstrated that the CFA models were able to estimate a factor correlation in each case and that these correlations were greater than or equal to the corresponding Pearson correlation in magnitude. This finding is consistent with the prior simulation study [9] and with established knowledge of how CFA models function. Specifically, because CFA methods assess the latent correlation between measures, and the correlation between latent variables is not attenuated by measurement error unlike PCCs [31-33], our results support the use of CFA to assess the relationship between a novel digital measure and a COA RM. The use of CFA in conjunction with PCCs facilitates a better understanding of the relationship that exists between the DM and the RM. CFA uses all available RM information in the analysis (ie, item-level data), versus PCCs and/or regression models alone, which aggregate the item-level RM data into total scores or mean values. Using multiple methods can lead to a range of estimates which can be used to support a validity argument.

However, the use of CFA comes with limitations. For example, CFA is known to require a larger sample size to produce stable estimates, and a number of necessary or sufficient conditions exist for the model to be identified, including requiring a minimum of 3 variables per factor (which implies that any COA RM used must comprise at least 3 items) [31,34,35]. While it is difficult to determine a uniformly applicable minimum sample size, the consensus is that a sample of participants in at least the hundreds is desirable [36]—a threshold that many AV studies for digital measures to date have not met [37-39]. With the improving feasibility and necessity of conducting observational research in the out-of-laboratory environment, larger sample sizes are increasingly accessible. Such research is likely to use COA-based RMs, making the CFA approach particularly relevant.

A range of relationship values was exhibited, which indicates both successful and unsuccessful model fits across the 4 real-world datasets. The performance of the measures shown in this work supports the feasibility of the selected statistical methods when implemented in real-world data, as their implementation here was successful despite the estimated values being weak. Importantly, the datasets used represented sDHTs from multiple domains, including smartphones/communication and actigraphy data, supporting the applicability of these methods across domains. It is possible that additional digital measurement approaches (such as speech, wearable electroencephalography, etc) may also be well-suited to leveraging the learnings of this work.

Reasons that weak relationships are observed may include the following: the study design is not optimized for the measure of interest, the chosen RMs are limited in their assessment of the underlying construct measured by the DM in a particular use environment, or a relationship simply may not exist. Notably, previous studies that have explored relationships between sDHTs (eg, step counts from wearables) and RMs such as the PHQ-9 have demonstrated low correlation magnitudes (eg, <|0.2|), suggesting that strong relationships may not necessarily be expected [40,41].

In the work conducted here, the datasets come from studies where the primary focus was not AV evidence generation. It is likely that this affected the estimation of relationships as the principles outlined in Textbox 1 were violated by each dataset in varying amounts.

### Recommendations

We recommend that investigators seek a high level of temporal coherence between the measures chosen for their AV study of a novel digital measure. Good temporal coherence means that the sDHT data used in the AV analyses aligns with the recall period of the COA-based RM. Poorer temporal coherence between measures may decrease the values estimated with agreement statistics because each individual’s level on the latent trait assessed by the measures (eg, health, disease severity, physical ability) may have changed over time. This is supported by the Brighten and mPower data, which have moderate to strong temporal coherence and the strongest relationships between measures.

In addition, we recommend that investigators seek a high level of construct coherence. Construct coherence assures that the DM and the RM are assessing as similar a concept as possible. Poor construct coherence is likely to lead to weak relationships between measures, even when using appropriate statistical methods. This is supported by the mPower data, which has the clearest and strongest construct coherence between measures and exhibited the strongest relationships between the measures.

We emphasize the need to determine the extent of data missing information and reduce measurement error in both the DM and RMs whenever possible. Data missing information particularly affects regression models, where incomplete cases will lead to entire participants’ data being excluded, thus reducing the sample size. This is supported by the mPower data, which retained its large sample size during analysis due to the data completeness of the RM. The R^2^ values in this dataset were two to five times stronger in general than in the Brighten study, which had substantial RM missing information in a smaller starting sample.

In line with the above methodological considerations, we encourage investigators to carefully plan their AV studies to avoid making incorrect inferences from their results. As always, an argument for validity should be constructed and presented to all stakeholders for advice, including regulators.

Finally, we recommend that investigators review the assumptions and requirements of the statistical methods they plan to use in the AV study to understand how assumption violations may distort their results and whether such violations are likely to occur. For example, while Pearson correlation is known to be relatively robust in terms of violations of parametric assumptions [42], CFA can be affected by moderate violations of its model assumptions [43,44], which can then affect fit index estimation, particularly in the case of the RMSEA model fit index [45].

### COA-Specific Recommendations

If an investigator is using COA-based RMs in their study, then we recommend longitudinal data collection, including using at least 1 RM with a daily recall period. Using a daily recall RM when the digital measure collects daily summary data is particularly recommended due to the expected strong temporal coherence between the measures.

When using RMs with multi-day recall periods, researchers should collect digital measure data on each day that the recall period pertains to and have a strong, enactable strategy to minimize data missing information in this period (such as calling patients the day before the beginning of the wear period to remind them to use the sDHT). These good practices can ensure the best opportunity for temporal coherence.

In addition, we recommend seeking construct coherence at the item level of the RMs. COA-based RMs are often derived from multidimensional clinical scales [46,47], which means that items or domains of a COA may have varying construct coherence with the DM. It may be appropriate to select specific items or domains that tightly reflect the latent construct under examination to use as an RM. This may lead to a stronger relationship between measures than a simple aggregation of all items or domains.

Table 15 summarizes all the above recommendations and provides practical directions to aid in appropriate study design for AV of novel digital measures.

**Table 15.** Considerations for designing a strong AV study for a novel digital measure.

Category	Considerations
Digital measure data collection	
Number of days	Longitudinal collection on consecutive days allows for the use of CFA methods, as long as at least 3 days are collected. Have an enactable participant engagement strategy to minimize data missing information.
Study design	
Rigor and quality of RMs	High-quality and high-rigor RMs enable the possibility for the strongest claims about the DM (see Bakker et al [5] for a potential hierarchy of RM quality and rigor).
Objectivity of RMs	Standardized data collection in an RM improves accuracy by reducing measurement error. Standardized data processing and standardized and trained interpretation reduce ambiguity and avoid issues with inter-rater variability.
RM construct coherence	Good construct coherence between measures may strengthen the values estimated from agreement statistics. Poor construct coherence may cause issues, even if the methods are well suited to assessing agreement. Consider the effect of construct coherence at the item and instrument level if using a COA RM.
RM temporal coherence	Good temporal coherence aligns data capture, meaning the measures assess a subject over the same period. Poor temporal coherence may decrease the values estimated with agreement statistics because the measures assess the construct at different times and the level of the construct is subject to change. If using a COA RM, Consider the benefit of using a daily recall period and assessing on the same days as the digital measure, if, for example, the digital measure collects daily summary count data. If using a multiday recall period COA, then applying the RM at the end of the period of digital measure data collection and collecting digital measure data on each day of the recall period are expected to increase temporal coherence.
Miscellaneous	To minimize distortion of results, review the assumptions and requirements of the statistical methods used and avoid violations of assumptions where possible.
	Identify factors that may influence missing information and measurement error in data capture and seek to minimize these where possible.
	Qualitatively assess the limitations of the study design ahead of conducting it and accept that the threshold for good agreement between measures may be smaller when well-established and rigorous RMs are not available.
	Consider more extensive clinical validation and validity testing by assessing repeatability, reliability, and ability to detect change over time when it appears the AV study will not allow you to establish rigorous validation claims. All claims must be validated and verified and backed up with sufficient evidence (subject to regulatory review).
	The quality of an RM affects what claims can be made about the performance of the DM. Perfect agreement between measures may not be enough for the validation of a novel DM, when the measure is hoped to outperform the RM and available RMs are poor.
Statistical methods for assessing agreement	
CFA	CFA can account for measurement error and variance at the item level when working with COA RMs since it can assess the latent correlation between the measures, and correlation between latent variables is not attenuated by measurement error.
Pearson correlation	Pearson correlation is stable, easier to compute, and relatively robust in terms of violations of parametric assumptions. Pearson correlation is known to underestimate the true correlation between measures because of attenuation by measurement error.
Linear regression	If multiple RMs are being used in the study, then MLR may provide a route to a stronger assessment of agreement between measures than individual SLR, particularly if one has an RM that captures daily data.
Sample size	The statistical methods used in an AV study affect the appropriate minimum sample size. Methods such as CFA often require a large sample, which could be fulfilled by repeated measures from each participant.

### Conclusions

This study demonstrated the feasibility of applying the analytical methodologies that were evaluated in our previous simulation study [9] to a series of real-world datasets. Furthermore, we demonstrated that the performance of different statistical tools (eg, CFA vs PCC) when applied to real data largely recapitulated the trends seen in previous simulated data [9]. Additionally, characteristics of the analyzed datasets, such as sample size, temporal coherence, and missing information patterns, had impacts on analysis that motivated our recommendations for specific design considerations in AV studies.

By using a standardized methodology for evaluating novel digital measures, developers, biostatisticians, and clinical researchers will be able to navigate the complex validation landscape more easily, with more certainty, and with more tools at their disposal when undertaking an analytical validity study.

Adopting standardized practices for the conduct of analytical validation studies creates a common approach that improves understanding and expedites the pathway to validation and regulatory review. This may, in turn, provide indirect cost savings in clinical trials by enabling a more rigorous development of sDHT-based technologies, which themselves offer considerable direct reductions in costs associated with recruitment, retention, and follow-up [48].

## Supplementary material

10.2196/70314 Multimedia Appendix 1 Description of datasets.

10.2196/70314 Multimedia Appendix 2 Description of statistical analysis methods.

## Abbreviations

AV: analytical validation
CFA: confirmatory factor analysis
COAs: clinical outcome assessments
DM: digital measure
IRB: institutional review board
MLR: multiple linear regression
RM: reference measure
sDHT: Sensor-based digital health technology
SLR: simple linear regression
