# Supplementary material for: Methods for Analytical Validation of Novel Digital Clinical Measures: Implementation Feasibility Evaluation Using Real-World Datasets
Source: J Med Internet Res. 2025 Nov 17;27:e70314. doi: 10.2196/70314 (PMC12622859; doi:10.2196/70314)
Supplement: Multimedia Appendix 1 [file jmir-v27-e70314-s001.docx]

# Appendix 1 - Description of data sets

Urban Poor dataset

The Urban Poor dataset arises from the study “The Economic Consequences of Increasing Sleep Among the Urban Poor”. The study used actigraphy to measure sleep objectively, and undertook a randomized three-week treatment providing information, encouragement, and improvements to home sleep environments in order to study each treatment’s effects on cognition, productivity, decision making, well being, and labor supply of low-income adults in Chennai, India.[[10][11]](https://paperpile.com/c/q6NelI/8BMq6+Ws1MM)

In this dataset, the variable “Number of awakenings during an entire night”, a measure with sleep quality as its underlying construct, was selected as the digital measure for assessment. Four measures of psychological well-being were chosen as reference measures: The Patient Health Questionnaire-9 (PHQ-9),[[15]](https://paperpile.com/c/q6NelI/D6gTy) Generalized Anxiety Disorder Questionnaire (GAD-7),[[17]](https://paperpile.com/c/q6NelI/D63tV) Rosenberg Self-Esteem Scale (Rosenberg),[[16]](https://paperpile.com/c/q6NelI/CPnc7) and a daily single-item patient global impression of happiness.[[11]](https://paperpile.com/c/q6NelI/Ws1MM) The PHQ-9, GAD-7 and Rosenberg were all collected at baseline. In total, 452 subject records in the study had data for all measures investigated in this work.

The multi-day recall reference measures were collected at baseline, indicating poor temporal coherence with the digital measure. The poor coherence is further evidenced by the interventions that were applied during the study period, creating a potential change in the state of the underlying construct being assessed.

Furthermore, the reference measures exhibit poor construct coherence with the digital measure: the reference measures are measures of psychological well-being, as opposed to the digital measure being a measure of sleep quality. Whilst sleep can be assumed to be somewhat affected by depression, anxiety and self-esteem, this is only tangential coverage of the concept of interest.

STAGES dataset

The STAGES dataset arises from the Stanford Technology Analytics and Genomics in Sleep (STAGES) study - a prospective cross-sectional, multi-center study of over 30,000 participants, undertaken to develop the critical infrastructure, tools and data for future sleep and sleep disorder research. Sleep and other surveys, polysomnography, and at least two weeks of actigraphy data were collected, along with other data such as biological samples and medical imaging.[[10]](https://paperpile.com/c/q6NelI/8BMq6)

In this dataset, daily step count (captured from actigraphy), a measure with physical activity as its underlying construct, was selected as the digital measure for assessment. Four measures, assessing a variety of constructs (fatigue, psychological well-being, breathing ability), were chosen as reference measures: The Fatigue Severity Scale (FSS),[[18]](https://paperpile.com/c/q6NelI/Y6gu1) PHQ-9[[15]](https://paperpile.com/c/q6NelI/D6gTy), GAD-7[[17]](https://paperpile.com/c/q6NelI/D63tV), and the Nasal Obstruction Symptom Evaluation (NOSE).[[19]](https://paperpile.com/c/q6NelI/lJGpy) Survey data were collected at inconsistent times during the study with respect to the actigraphy data collection. In total, 964 subject records in the study had data for all the chosen measures.

The survey data were collected at inconsistent times during the study: usually this was near in time to polysomnography collection, but often at very different times to the actigraphy data collection. As such, the reference measures exhibited poor temporal coherence with the digital measure. Furthermore, the reference measures exhibit poor construct coherence with the digital measure: the digital measure is a measure of physical activity, whereas the reference measures are measures of psychological well-being, breathing obstruction and fatigue. While it can be assumed that these constructs are somewhat linked to a participant’s physical activity abilities, this link is likely to be only tangential.

mPower dataset

The mPower dataset arises from the mPower study[[12]](https://paperpile.com/c/q6NelI/Fw4OA) - a study piloting new approaches to the monitoring of key indicators of Parkinson Disease progression and diagnosis by comparing well-established subjective symptom measurements with novel sensor-based metrics obtained from smartphones. The goal of this study was to understand the dynamic variability of patient symptoms, and how these variations may arise and modulate, to aid in the development of personalized interventions.

Participants in the study were presented with four smartphone activities to be completed on a daily basis. Of the four activities undertaken, the number of smartphone screen taps recorded from the daily tapping activity was chosen as the digital measure. The underlying construct of this measure is fine motor control in Parkinson’s disease. Two PROs were chosen as the reference measures: a selected subset of questions from MDS-UPDRS[[20]](https://paperpile.com/c/q6NelI/yeQ6l) and the PDQ-8;[[21]](https://paperpile.com/c/q6NelI/LzAku) both of which assessed the underlying construct of functioning whilst suffering from Parkinson’s disease. In total, 1641 subject records had data for the digital measure and both reference measures.

The reference measures exhibited a strong temporal coherence with the digital measure: we were able to isolate digital measure data from the recall period of both reference measures, with minimal missing data in the subject records. Construct coherence of the reference measures with the digital measure was also moderate to strong: the digital measure and the reference measures were measures of Parkinson’s Disease. Unlike the digital measure, the reference measures did not solely focus on the motor skills of the subjects, additionally targeting the way a Parkinson’s disease patient feels and functions in their daily life, which is the reason we consider the construct coherence only moderate to strong.

Brighten dataset

The Brighten dataset comprises two studies funded by the National Institute for Mental Health, with the objective to document and compare use patterns and clinical outcomes across the United States between three different self-guided mobile apps for depression. The goal of the work was to create insights that have long term potential to predict and mediate future depression-related concerns.[[13]](https://paperpile.com/c/q6NelI/kUSoB) Passive smartphone communications data were collected, alongside passive mobility and weather data and a variety of survey data.

Using the passive smartphone communications data, three variables with psychological well-being as their underlying construct (“Unique numbers calls incoming”, “Unique numbers calls outgoing” and “Unique numbers texts received”) from the second study were selected as the digital measures for assessment. Two measures of psychological well-being were chosen as reference measures: the PHQ-9[[15]](https://paperpile.com/c/q6NelI/D6gTy), assessing a two-week recall period, and the PHQ-2, a 2-item global impression of severity administered daily.[[22]](https://paperpile.com/c/q6NelI/U1ibH) Digital measure data and PHQ-2 data for each subject record were collated from the week preceding the administering of the PHQ-9. Subject records with completely missing data for any of the measures were excluded entirely. Each question from the PHQ-2 was treated as a separate reference measure. In total, 89 subject records from the full study population had data suitable for analysis.

The reference measures exhibited a moderate to strong temporal coherence with the digital measure: we were able to isolate digital measure data from the recall period of the PHQ-9, although there was substantial missing data in some of the subject records. Construct coherence of the reference measures with the digital measure was moderate to weak: whilst depression can be expected to have an effect on a person’s willingness to engage in communication with others, this would be expected to be a weak correspondence. Furthermore, a person’s normal behavioral habits are not adjusted for: for example, if a person regularly uses their phone for work as a self-employed tradesperson, their communications volume would likely be greater than an average person, even accounting for a decrease as a result of strong depression symptoms.
