# Supplementary material for: Methods for Analytical Validation of Novel Digital Clinical Measures: Implementation Feasibility Evaluation Using Real-World Datasets
Source: J Med Internet Res. 2025 Nov 17;27:e70314. doi: 10.2196/70314 (PMC12622859; doi:10.2196/70314)
Supplement: Multimedia Appendix 2 [file jmir-v27-e70314-s002.docx]

# Appendix 2 - Description of statistical analysis methods

### Pearson Correlation

In each dataset, the linear correlation between the digital measure and each multi-day recall reference measure was determined by calculating the Pearson Correlation Coefficient (PCC). Specifically, for each study participant: their scaled total score from one application of the multi-day recall reference measure was paired with the mean value of their seven days of digital measure data isolated during the recall period for that reference measure. This was repeated for each application of the reference measure, and each study participant. The Pearson correlation between the resulting two sets of data was then calculated.

### Regression models

In each dataset, a simple linear regression (SLR) model was built for each multi-day recall reference measure, by using the two sets of data explained above. More specifically, the reference measure total score data was used as the predictor variable, and the means of the digital measure data was used as the outcome variable.

In each dataset, multiple linear regression (MLR) models were built using each combination of multi-day recall measures available as predictor variables. For example, in the Urban Poor dataset, data was available for three multi-day recall measures: PHQ-9, GAD-7, and Rosenberg. Thus, four MLR models were built: PHQ-9 and GAD-7, PHQ-9 and Rosenberg, GAD-7 and Rosenberg, and all three RMs. As in the SLR models, the reference measure total scores data was used as the predictor variables, and the means of the digital measure data as the outcome variable.

Where daily recall reference measures were available (in the Urban Poor and Brighten datasets), MLR models were created for every combination of daily and multi-day recall reference measures as the predictor variables, with the mean digital measure data remaining as the outcome variable. Daily reference measures were included as predictors in two ways: firstly as a single predictor using the mean values for each subject record, and secondly as separate predictors with the individual days of data included separately.

For each regression model created (either SLR or MLR), the appropriate R^2^ statistic was calculated: for SLR models this was the standard R^2^ statistic; for MLR models this was the adjusted R^2^ statistic

${\overline{R}^{2}= 1 - (1-R^{2})\frac{n-1}{n-p-1}}$

where n is the number of observations and p is the number of reference measures.

### Confirmatory Factor Analysis

A two-factor CFA model with correlated factors was created for each combination of digital measure variable and multi-day recall reference measure. In each model, the individual items of the reference measure were loaded onto a “reference measure” factor, and the individual days of digital measure data were loaded onto a “digital measure” factor (Figure S1). Each model used pairwise deletion to account for data missingness and unweighted least square as the estimator.


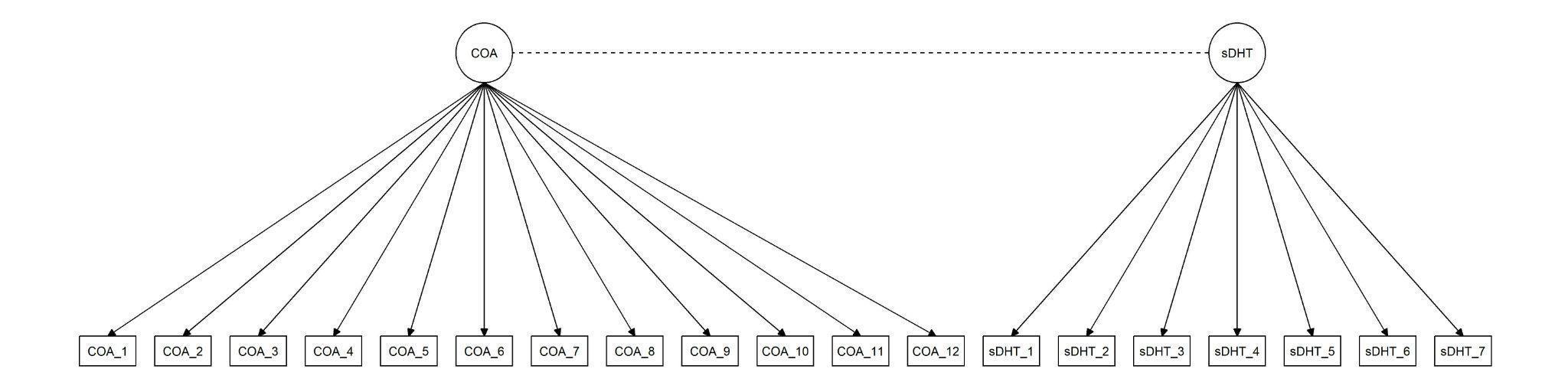


**Figure S1 - Path diagram for the two-factor CFA model**

The model-implied factor correlation (that is, the standardized value of the factor covariance) between the reference measure and digital measure factors was calculated, and four model fit statistics were computed: Comparative Fit Index (CFI), Tucker-Lewis Index (TLI), Root Mean Square Error of Approximation (RMSEA), and Standardized Root Mean Square Residual (SRMR). The fit statistics were evaluated against the following thresholds to determine if each model was an acceptable fit to the data: [[26][27]](https://paperpile.com/c/q6NelI/GoSm+Tl4K)

- CFI and TLI acceptable fit: values ≥ 0.90.
- RMSEA and SRMR acceptable fit: values < 0.08.
